# Real-World Safety Data of the Orphan Drug Onasemnogene Abeparvovec (Zolgensma^®^) for the SMA Rare Disease: A Pharmacovigilance Study Based on the EMA Adverse Event Reporting System

**DOI:** 10.3390/ph17030394

**Published:** 2024-03-19

**Authors:** Rosanna Ruggiero, Nunzia Balzano, Maria Maddalena Nicoletti, Gabriella di Mauro, Federica Fraenza, Maria Rosaria Campitiello, Francesco Rossi, Annalisa Capuano

**Affiliations:** 1 Department of Experimental Medicine, University of Campania “Luigi Vanvitelli”, 80138 Naples, Italy; nunziabalzano95@gmail.com (N.B.); gabriella.dimauro@unicampania.it (G.d.M.); annalisa.capuano@unicampania.it (A.C.); 2 Pharmacovigilance and Pharmacoepidemiology Regional Center of Campania Region, 80138 Naples, Italy; 3 Department of Precision Medicine, University of Campania “Luigi Vanvitelli”, 80138 Naples, Italy; maddalenanicoletti9@gmail.com; 4 UOC Pharmacy, AORN Santobono-Pausilipon Children’s Hospital, 80122 Naples, Italy; 5 Department of Obstetrics and Gynaecology and Physiopathology of Human Reproduction, ASL Salerno, 84124 Salerno, Italy; 6 Department of Life Sciences, Health and Health Professions, Link Campus University, 00165 Rome, Italy

**Keywords:** gene therapy, orphan drug, safety data, pharmacovigilance, spinal muscular atrophy

## Abstract

The recent introduction of the innovative therapy, onasemnogene abeparvovec (Zolgensma^®^), has revolutionized the spinal muscular atrophy (SMA) therapeutic landscape. Although Zolgensma^®^ therapy has proven to lead to functional improvements in SMA children, some gaps in its safety profile still need to be investigated. To better characterize the Zolgensma^®^ safety profile, we conducted a retrospective observational study, analyzing all the Individual Case Safety Reports (ICSRs) referred to it and collected in the European pharmacovigilance database between 1 January 2019 and 22 September 2023. We found 661 ICSRs related to Zolgensma^®^, with a growing trend in the annual reporting. The majority of the reports were sent by healthcare professionals and referred to infant females. In more than 90% of the cases, Zolgensma^®^ was the only reported suspected drug. Out of a total of 2744 reported ADRs, increased hepatic enzymes, pyrexia, vomiting, and thrombocytopenia were the most commonly reported adverse reactions. Of these adverse reactions (ADRs), 56.9% were serious, causing or prolonging the patient’s hospitalization. A total of 39 ICSRs related to cases with a fatal outcome. Alterations in the heart rhythm, acute hepatic failure, and hepatic cytolysis emerged among the cardiac and hepatic disorders, respectively.

## 1. Introduction

Spinal muscular atrophy (SMA) is a leading genetic cause of infant mortality [1]. It is a rare neuromuscular disease, with an estimated incidence of approximately 1 in 10,000 live births and a prevalence of 1–2 in every 100,000 people [2]. Muscle weakness and atrophy, particularly affecting the lower limbs and respiratory muscles, are the typical SMA symptoms. In the most serious forms, this evolves into reduced or absent abilities to move, swallow, and breathe [3]. The progressive degeneration of the α-motor neurons in the spinal cord and defects in neuromuscular junction development are characteristic of SMA and are due to the reduced expression or deficiency of the survival motor neuron (SMN) protein [4]. This ubiquitous protein is essential for motor system and motoneuron functionality [1]. SMN is coded by two genes mapped to chromosome 5q13: the telomeric *SMN1* and the centromeric *SMN2* [4]. The genetic cause of SMA has been identified as deletions or mutations in the *SMN1* gene, the only gene able to code for a fully functional SMN protein. The *SMN2* gene mainly produces an SMN protein with diminished functions, known as SMNΔ7. This truncated isoform, representing 85–90% of *SMN2* products, is highly unstable and more susceptible to degradation by the ubiquitin–proteasome pathway. Only 10–15% of *SMN2*-coded product is a functional protein. The age of symptom onset and the severity of clinical course in SMA are inversely correlated with the *SMN2* copy number [5]. Based on these three correlated variables, SMA is categorized into five types, from the prenatal-onset and fatal one (type 0) to adult-onset and less invaliding form (type IV). However, there are degrees of severity even within an individual type, and as many as 25% of patients elude precise classification [6]. The infantile form (type I SMA) is the most frequent, accounting for approximately half of patients. Children with type I SMA show the following symptoms in the first 6 months of life: hypotonia, delayed motor milestones, feeding difficulties, and never sitting independently [7]. Moreover, infants with type I SMA usually develop respiratory failure before 2 years of life, requiring permanent ventilation and nutritional support [6]. Regarding the therapeutic possibilities, until 2016, the therapy only supported vital functions. The identification of the molecular mechanisms underlying SMA’s onset and progression allow us to identify some SMN-dependent or -independent therapeutic strategies. To date three SMA treatments are available, all aimed at increasing SMN protein levels in different ways [8]. In particular, both the antisense oligonucleotide nusinersen (Spinraza^®^) and risdiplam (Evrysdi^®^) work by changing SMN2 splicing, while onasemnogene abeparvovec (Zolgensma^®^) works by replacing the SMN1 gene [9].

Zolgensma^®^ was the first SMA gene therapy approved by the Food and Drug Administration (FDA) and the European Medicine Agency (EMA)—in 2017 and 2020, respectively [9]. The recent introduction of this innovative therapy revolutionized the SMA therapeutic landscape [10]. To date, Zolgensma^®^ is indicated for the treatment of patients with 5q SMA with a bi-allelic mutation in the SMN1 gene and a clinical diagnosis of SMA type 1, or patients with 5q SMA with a bi-allelic mutation in the *SMN1* gene and up to three copies of the *SMN2* gene [9]. The used AAV9 vector delivers a functional copy of the *SMN1* gene to the motoneuron nucleus, as a primary source of the functional SMN. In addition to the innovative mechanism of action, the great innovation of Zolgensma^®^ is the method of administration, consisting of a single intravenous infusion over 60 min.

This one-shot and lifetime gene replacement therapy directly works on the monogenic cause of SMA [11]. This feature represents a huge step forward compared with the pharmacological alternatives nusinersen, which requires intrathecal administration, and risdiplam, which needs daily oral administration. Even if Zolgensma^®^ has been proven to lead to functional improvements in treated SMA children, reducing pulmonary and nutritional support requirements as well as hospitalization rate and improving motor function [12], some gaps in its safety profile still need to be investigated [9,10]. There are still some unanswered questions that need to be addressed: the long-term benefits remain to be determined and the pre-approval safety data need to be confirmed/refused in a real-world setting. Moreover, its cardiac toxicity, as well as the dorsal root ganglion toxicity and the tumorigenicity due to chromosomal integration are important potential risks that require investigation [13]. 

In this context, analysis of post-marketing data is particularly important as a source of new information that has not yet emerged, especially in specific populations [14]. In light of this, the aim of this study was a post-marketing evaluation of the safety profile of Zolgensma^®^ through the analysis of data retrieved from the European pharmacovigilance database Eudravigilance (EV).

## 2. Results

**Figure 1:**
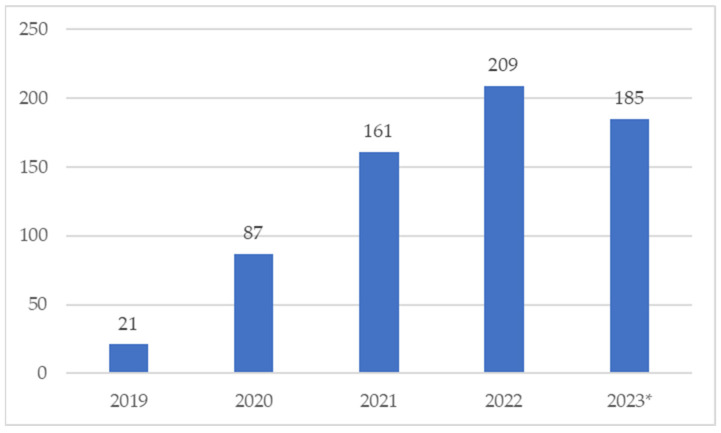
Distribution of Individual Case Safety Reports (ICSRs) reporting onasemnogene abeparvovec (Zolgensma^®^) as a suspected drug by year (2019–2023). * Up to 22 September 2023.

During our study period, 661 ICSRs related to the use of onasemnogene abeparvovec were collected into the EV, describing 2744 suspected adverse reactions (ADRs). Starting from 2019, a constant increase in ICSR reporting was observed, with a peak of 209 ICSRs sent in 2022, as shown in Figure 1.

The demographic characteristics of the patients are described in Table 1. Considering the distribution of ICSRs for age groups, a higher proportion (*n* = 396; 59.9%) emerged for the infants group (2 months–2 years), followed by children (3–11 years), which comprised 76 ICSRs (11.5%). Neonates were represented in 9.2% of total ICSRs (*n* = 61). Among all cases, 287 ICSRs (43.4%) were related to female patients, while males accounted for 249 ICSRs (37.7%). In 125 ICSRs the patient sex was not specified. Healthcare professionals were the most represented reporter type, as they submitted the majority of onasemnogene-related ICSRs (88.5%) collected in the EV. The majority of the ICSRs reported only onasemnogene as a suspected drug (92.1%), with no concomitant drugs reported in 61.6% of cases. Prednisolone and nusinersen were the other suspected drugs more represented, described in 26 (35.1%) and 23 (31.1%) ICSRs, respectively. Risdiplam was indicated as suspected drug in only one case. All the other suspected drugs and their therapeutic indication are reported in Supplementary Table S1.

Regarding the seriousness distribution of ADRs, 43% were not serious (*n* = 1185), and more than 50% of reported adverse events were classified as serious. In particular, serious ADRs were mainly categorized as other medically important conditions (*n* = 721; 26.3%). Moreover, serious adverse events caused/prolonged the patient’s hospitalization or were life-threatening in 21% (*n* = 588) and 4.3% (*n* = 118) of the cases, respectively. Although in 54.7% of the cases the ADR outcome was unknown (*n* = 1502), the most reported outcomes were favorable, resulting as recovered/resolved (*n* = 676; 24.6%) or recovering/resolving (*n* = 238; 8.7%). On the other hand, 6.9% of ADRs did not resolve (*n* = 188) and a fatal outcome occurred for 130 out of all reported ADRs (4.7%) (Table 2). The 130 ADRs with the fatal outcome were described in a total of 39 ICSRs, mainly related to patients aged 2 months–2 years (*n* = 33). Only one fatal case was related to a patient aged 0–1 month and no fatal cases were related to the children’s group (3–11 years). Three ICSRs with fatal outcomes included nusinersen as another suspected drug. We have reported all adverse events with fatal outcomes in Supplementary Table S2. Fatal outcomes were mainly due to cardiac arrest (*n* = 8; 6.2%), respiratory arrest or respiratory failure (both *n* = 5; 3.8%), or acute hepatic failure (*n* = 4; 3.1%).

In Table 3 we describe the ADRs most frequently reported, primarily resulting in pyrexia (*n* = 173; 6.3%), vomiting (141; 5.10%), increased aspartate and alanine aminotransferase (*n* = 129; 4.70% and *n* = 120; 4.40%), and thrombocytopenia (*n* = 118; 4.30%). In Table 3 we also compare the single reported ADRs with the total number of ICSRs. However, by adding those ADRs which were indicative of the same clinical adverse event but reported differently by the reporters with different terms, we found that 505 ADRs indicated an increase in hepatic enzyme, 195 ADRs indicated an increase in body temperature, and a decrease in count platelet was described 167 times, accounting in 76.40%, 29.50%, and 25.26% of the ICSRs, respectively.

**Figure 2:**
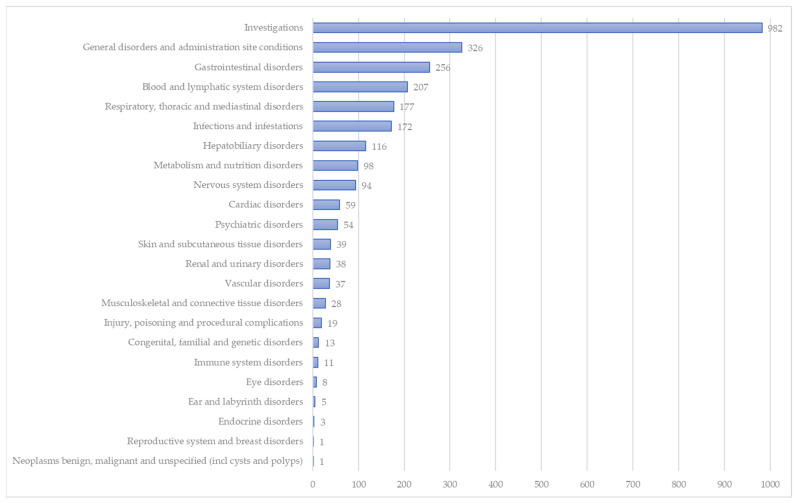
Distribution of reported adverse Zolgensma^®^-related reactions categorized as System Organ Classes (SOCs).

In Figure 2, the reported adverse events are categorized in System Organ Classes (SOCs). Of these SOCs, “Investigations” (35.8%), “General disorders and administration site condition” (11.9%), “Gastrointestinal disorders” (9.3%), “Blood and lymphatic system disorders” (7.5%), and “Respiratory, thoracic and mediastinal disorders” (6.5%) were more frequently reported.

Among the events belonging to the investigation SOC (Table 4), the most common ones indicated qualitative results of the conducted hepatic, hematopoietic, and cardiac clinical laboratory tests. In particular, increased hepatic values, like aspartate and alanine aminotransferase (*n* = 129 and *n* = 120) and gamma-glutamyl transferase (*n* = 23), as well as blood bilirubin (*n* = 11) or generic liver function tests increased (*n* = 35), accounting for more than 40% of the investigations. The decrease in platelet count (*n* = 49) and the increase in monocyte count (*n* = 13) emerged as hemopoietic disturbances. Other alterations, such as increased values of blood lactate dehydrogenase (*n* = 22) and troponin I (*n* = 40) and T (*n* = 15), were signs of tissue damage. Finally, alterations in the heart rhythm, acute hepatic failure, and hepatic cytolysis emerged among the cardiac and hepatic disorders, respectively, as described in Table 5 and Table 6. Regarding the neoplasms (benign, malignant, or unspecified (including cysts and polyps)) SOC, only one ICSR reported an adverse event belonging to this category. This was an astrocytoma malignant occurrence in a male patient in the 2 months–2 years group, which caused or prolonged his hospitalization, but for which the outcome was not available.

## 3. Discussion

Our study aimed to describe the Zolgensma^®^ safety profile emerging from a real-world context by analyzing data collected in the European pharmacovigilance database. Our choice was based on the consideration that, even if pre-marketing clinical trials allow the acquisition of the majority of important drug-safety data, only use in a real-world context can better define the safety aspects, allowing for the identification of rarer ADRs [15,16,17,18]. For these reasons, continuous post-marketing monitoring of drug safety is essential. Long-term safety evaluation is always considered necessary, especially for drugs that have been authorized with conditional and/or accelerated approvals, as often happens for innovative drugs [19,20,21,22]. This necessity is amplified for some particular drug classes, like the orphan ones used for rare diseases [23]. Zolgensma^®^ meets all these mentioned characteristics, as an innovative drug, supported through EMA’s PRIority MEdicines (PRIME) scheme [24], which received a conditional marketing authorization for the rare disease, SMA [24]. The efficacy and safety data obtained in post-marketing can be particularly important for possible confirmation or re-evaluation of a drug’s safety and efficacy profiles and innovativeness, as well as its price [25,26]. Also, this aspect is particularly relevant for Zolgensma^®^, considering its high cost (approximately $2 million per course of treatment) [27]. However, some pharmacoeconomic studies in the literature support the cost-effectiveness of this gene therapy compared with the other therapeutic possibilities, when used in pre-symptomatic patients [28,29,30,31,32]. 

From our analysis, 661 safety reports related to Zolgensma^®^ and collected in the European pharmacovigilance database emerged. Excluding the partial data referred to 2023, the annual reporting trend was growing up to 2022. Our reporting trend differed compared with the trend that emerged from a recent study conducted on the US pharmacovigilance database [33]. Following the surge in the Zolgensma^®^-related ADR reporting in 2020 compared with 2019, the authors found a decreasing trend in the number of reports collected until 2022 in the US database, the Food and Drug Administration Adverse Event Reporting System (FAERS) [33]. Regarding the sex distribution, although some evidence suggests that males may be more vulnerable to SMA than females [34,35], our analysis revealed a greater reporting of ADRs in female patients. This result, as well as the major distribution in terms of the reporter type in favor of HPs, is in line with the analysis conducted by Zhuang et al. on the US database [33]. As described in other studies, HPs represent the major reporting source, even if, to date, citizens can also send safety reports to regulatory authorities [36,37]. The distribution of reports by age group of patients is also comparable, although not completely coincident. Our analysis revealed that the majority of reports referred to patients aged between 2 months and 2 years, while the analysis of FAERS data revealed that the majority of adverse events reported for onasemnogene abeparvovec referred to patients less than 1 year of age. However, this slight discrepancy between the two results could be traced back to the different methods of data extraction and categorization related to the age of the patients. The data extraction method used for the present pharmacovigilance study did not allow us to trace the precise age of the patient, but only the age group to which it referred.

As regards the types of adverse events, our results were in line with the recently published Zhuang et al. study [33]. Both pharmacovigilance studies highlighted a higher reporting of pyrexia, vomiting, increased aspartate aminotransferase, increased alanine aminotransferase, thrombocytopenia, increased transaminases, and increased liver enzymes, as ADRs related to the SMA gene therapy. Hepatotoxicity, transient thrombocytopenia, and thrombotic microangiopathy have been identified as possible Zolgensma^®^ toxicities [13]. These safety aspects emerged among the top twenty ADRs reported in our dataset. In addition to possible liver damage, signs of cardiac damage also emerged among the most frequently adverse events reported, such as the increase in troponin I. The heart and the liver had also been found to be the main target organs of Zolgensma^®^ toxicity in pre-clinical studies [38]. According to the main safety results derived from non-clinical studies, inflammation, edema, fibrosis, and characteristics of widespread myocardial degeneration/regeneration in the ventricles emerged at the cardiac level in mice. These findings were present at all studied doses and were dose-related in terms of seriousness [38].

As regards a possible hepatotoxicity risk, our results confirmed the possible hepatic damage evidenced by increased hepatic enzymes or tests (ALT and AST). This safety aspect, already found in pre-clinical studies [38], emerged from the first patients enrolled in pre-marketing clinical trials [39]. In post-mortem tissue samples from two patients treated with onasemnogene abeparvovec, the highest concentrations of vector DNA were found in the liver [39]. Safety signals have also emerged in the post-marketing context about the liver and heart organs [38]. Similarly, our analysis revealed reports of possible liver damage (hypertransaminasemia, liver disorder, acute liver failure, and liver cytolysis) and cardiac rhythm alterations. The increase in hepatic enzymes can be caused by the immune response induced the gene therapy [40]. Both hepatobiliary and hematologic abnormalities can be considered the immune response to the viral capsid. Moreover, hepatoxicity risk is complicated by the major predisposition of patients with SMA to acute liver injury [39]. Abnormal fatty acid metabolism, as the reported cause of liver failure, was reported in children with SMA [40]. Their increased risk of dyslipidemia and fatty liver could predispose them to hepatotoxicity [41]. Zolgensma^®^-related hepatoxicity can generally be mitigated with prophylactic prednisolone. This latter emerged among the most frequently reported other concomitant drug in our study. These hepatic and heart rhythm disorders also emerged from the analysis of the US FAERS pharmacovigilance database. Furthermore, it is important to note that, in both studies, a risk of damage to the heart was highlighted, evidenced by an increase in troponin blood concentrations. According to Zhuang et al., such increased levels of troponin I and T have been identified as idiosyncratic adverse reactions to onasemnogene abeparvovec [33]. However, the possible causal relationship and the biological plausibility between onasemnogene abeparvovec treatment and cardiotoxicity is still unclear. An increased risk of cardiac events may be related to SMA itself. Out of all the suspected ADRs reported, some may be symptoms of progression of SMA, as a disease with a very broad and subjective spectrum of symptoms, which also change based on the type of SMA. Heart and liver function should be monitored before, during, and after use of the drug to prevent more serious manifestations. In addition to hepatic and cardiac adverse events, serious respiratory adverse events, such as bronchiolitis, pneumonia, respiratory distress, and respiratory syncytial virus bronchiolitis, were also observed in the STR1VE trial. However, they were considered unrelated to onasemnogene abeparvovec [41]. Pneumonia and dyspnea emerged among the top twenty ADRs in our analysis. Regarding the possible tumorgenicity due to chromosomal integration as a potential Zolgensma^®^ adverse event, only one case describing a tumoral adverse event emerged in our analysis. This potential risk certainly requires more long-term monitoring and careful analysis with more appropriate methodologies. The same applies to the hypothetical risk of dorsal root ganglion toxicity, which is more difficult to identify through pharmacovigilance database analysis. However, none of the retrieved ICSRs involved either areflexia or hyporeflexia, which have emerged in other studies as sensory abnormalities suggestive of gangliopathy [42]. 

Our study showed some strengths as well as some limitations. The use of a low-cost, broad database, coming from the real world setting, overcame several limitations of data collection during the clinical and pre-approval trials, and helped in characterizing drug safety profiles. However, this study also had several limitations, particularly the underreporting phenomenon, which characterizes all the spontaneous reporting systems [43,44,45,46]. This phenomenon can have repercussions for public health, interfering with the ADRs’ incidence quantification and risk estimates, as well as delaying the identification of safety signals. Only 6–10% of all adverse events are reported to regulatory authorities [43,47]. We cannot exclude the fact that some safety cases may not have been reported to the drug authorities and thus not collected in the EV. In addition to the underreporting, possible missing information can also be a difficulty in analysis based on spontaneous reporting systems. The quality of information reported may be incomplete and lack useful clinical information. This can delay a complete evaluation of confounding factors such as clinical history and concomitant comorbidities and medications. From our data source, we also could not retrieve information on the exact dates of administration and event onset—useful for a long-term safety evaluation. Moreover, our data source did not report the exact number of patients exposed to the treatment as real users of Zolgensma^®^; this would be necessary for reporting-rate evaluation. Considering these limitations, our study only aimed to analyze ICSRs related to Zolgensma^®^ and to show its adverse event characteristics; it refrained from asserting any direct causal association between the SMA gene therapy and the adverse events reported as suspected ADRs. Considering the peculiarities of the rare disease, SMA, further investigations with appropriate methodologies are needed. However, analysis of the post-marketing database often represents the first step or the primum movens in identifying new safety aspects needing deeper examination.

## 4. Materials and Methods

On 22 September 2023, we retrieved data on Individual Case Safety Reports (ICSRs) reporting Zolgensma^®^ as a suspected drug from the EV, the EMA pharmacovigilance database (www.adrreports.eu, accessed on 22 September 2023). This is a publicly available database, that contains the spontaneous safety reports submitted to the EMA [15]. Analysis of pharmacovigilance databases allows for early detection of safety signals and quantification of the association between drugs and reported adverse events (AEs) [16,17]. A wide variety of sources can send safety reports, both healthcare professionals (HPs) (e.g., physicians, pharmacists, and nurses) and non-healthcare professionals (patients, citizens, lawyers, and consumers) [18,19,20,21,22,23]. ICSRs reporting onasemnogene as a suspected drug were searched and a list of cases was exported. Our study period was 1 January 2019–22 September 2023. We checked for duplicates highlighted by the same ICSR code. We conducted an observational retrospective study, verifying the reporting annual trend, the distribution of the reports by sex and age group, the source country, and the reporter type, as well as the number of concomitant or suspected drugs. We analyzed the downloaded data. In the EV, the age groups are categorized as neonates, including preterm and term newborns (0–1 month), infants (2 months–2 years), and children (3–11 years). The reporter types are classified as healthcare professional and non-healthcare professional. The source country is not specified, but it is distinguished as belonging to the European Economic Area or the Non-European Economic Area. The suspected adverse drug reactions (ADRs) were analyzed in terms of seriousness, outcome, and type of event. According to current pharmacovigilance regulations, an ADR is categorized as serious if it induces death, hospitalization or prolongation of hospitalization, severe or permanent disability, threat to life, congenital abnormalities/birth defects, or is considered clinically relevant.

In the EV, the reported ADRs are coded according to the Medical Dictionary for Regulatory Activities (MedDRA). We analyzed ADRs based on Preferred Terms (PTs) and corresponding System Organ Class (SOC). We focused on the most representative SOC, and the events belonging to Hepatobiliary disorders and Cardiac disorders SOCs. Since each ICSR can contain more than one ADR, the total number of ADRs can be higher than the overall ICSR number. Moreover, cases with fatal outcomes were investigated as a sub-analysis, describing their distribution in terms of sex and age group, as well as the type of fatal event. All data manipulation and statistical analysis were performed using R Statistical Software (version 4.0.3).

## 5. Conclusions

Our study aimed to better define the safety profile of drugs, by using the spontaneous reports of safety data in routine clinical practice. The results of our study confirmed hepatoxicity as the principal issue emerging from Zolgensma^®^ use in clinical practice. Considering that SMA children are predisposed to liver dysfunction, hepatic monitoring is necessary during the treatment. In the same way, cardiac monitoring is also important, considering the possible cardiotoxicity induced by Zolgensma^®^, even if this latter requires more investigations to better define it and its plausible correlation to the treatment. From our analysis, alterations in the heart rhythm, acute hepatic failure, and hepatic cytolysis emerged among the cardiac and hepatic disorders, respectively. 

Approval of Zolgensma^®^ for the treatment of SMA has shown evident benefits. Nevertheless, some safety concerns remain unresolved, especially long-term ones. These latter include the potential dorsal root ganglion (DRG) toxicity and late-onset motor dysfunction that emerged as long-term AAV9-mediated SMN overexpression in mouse models [48]. In this context, systematic data collection and long-term follow-up could better define Zolgensma’s long-term safety profile. This is particularly important for the hypothetical tumorgenicity due to chromosomal interaction, which has been identified as an important potential risk. In conclusion, further studies and continuous monitoring are therefore necessary for SMA gene therapy, as an important and expensive therapeutic strategy for a rare disease. The systematic and spontaneous collection of post-marketing data is even more fundamental in the context of such rare diseases and orphan and innovative drugs such as Zolgensma^®^.

## Figures and Tables

**Table 1:** Demographic characteristics of Individual Case Safety Reports (ICSRs) having onasemnogene abeparvovec (Zolgensma^®^) as the suspected drug sent through the EV database from January 2019 to September 2023.

	Overall (*n* = 661)
Age Group	
0–1 month (neonates)	61 (9.2%)
2 months–2 years (infants)	396 (59.9%)
3–11 years (children)	76 (11.5%)
12–17 years (adolescents)	1 (0.2%)
18–64 years (adults)	1 (0.2%)
Not specified	126 (19.1%)
Patient Sex	
Female	287 (43.4%)
Male	249 (37.7%)
Not specified	125 (18.9%)
Reporter Type	
Healthcare professional	585 (88.5%)
Non-healthcare professional	76 (11.5%)
Country	
European Economic Area	319 (48.3%)
Non-European Economic Area	342 (51.7%)
Concomitant Drugs per ICSR	
0	407 (61.6%)
1	143 (21.6%)
2	49 (7.4%)
3	33 (5.0%)
4	12 (1.8%)
≥5	17 (2.6%)
Suspected Drugs per ICSR	
1	609 (92.1%)
2	42 (6.4%)
3	8 (1.2%)
4	1 (0.2%)
≥5	1 (0.2%)

**Table 2:** Characteristics of the suspected adverse drug reactions (ADRs) related to onasemnogene abeparvovec (Zolgensma^®^) sent to and collected in the European pharmacovigilance EudraVigilance database from 2019 to 22 September 2023.

	Overall ADRs (*n* = 2744)
ADR Seriousness Criteria	
Caused/prolonged hospitalization	588 (21.4%)
Disabling	2 (0.1%)
Life-threatening	118 (4.3%)
Not serious	1185 (43.1%)
Other medically important condition	721 (26.3%)
Results in death	130 (4.7%)
ADR Outcome	
Fatal	130 (4.7%)
Not recovered/not resolved	188 (6.9%)
Recovered/resolved	676 (24.6%)
Recovered/resolved with sequelae	10 (0.4%)
Recovering/resolving	238 (8.7%)
Unknown	1502 (54.7%)

**Table 3:** The top twenty adverse events reported as suspected adverse drug reactions (ADRs) related to onasemnogene abeparvovec (Zolgensma^®^) sent to and collected in the European pharmacovigilance EudraVigilance database from 2019 to 22 September 2023.

Reported Adverse Events	Overall ADRs (*n* = 2744)		Overall ICSRs (*n* = 661)
	*n*	% per Total ADRs	% per Total ICSRs
Pyrexia	173	6.30%	26.17%
Vomiting	141	5.10%	21.33%
Aspartate aminotransferase increased	129	4.70%	19.52%
Alanine aminotransferase increased	120	4.40%	18.15%
Thrombocytopenia	118	4.30%	17.85%
Transaminases increased	91	3.30%	13.77%
Hepatic enzyme increased	77	2.80%	11.65%
Decreased appetite	50	1.80%	7.56%
Platelet count decreased	49	1.80%	7.41%
Troponin I increased	40	1.50%	6.05%
Pneumonia	36	1.30%	5.45%
Liver function test increased	35	1.30%	5.30%
Hypertransaminasaemia	30	1.10%	4.54%
Asthenia	29	1.10%	4.39%
Dyspnoea	25	0.90%	3.78%
Gamma-glutamyltransferase increased	23	0.80%	3.48%
Blood lactate dehydrogenase increased	22	0.80%	3.33%
Body temperature increased	22	0.80%	3.33%
Nausea	21	0.80%	3.18%
Apathy	19	0.70%	2.87%
Thrombotic microangiopathy	19	0.70%	2.87%

**Table 4:** Adverse events reported in Zolgesma^®^-related ICSRs collected in the EudraVigilance spontaneous reporting system from 2019 to 22 September 2023, belonging to the Investigations MedDRA System Organ Class (frequency > 1.00%).

Adverse Events Belonging to the Investigation MedDRA SOC (*n* = 982)	*n*	%
Aspartate aminotransferase increased	129	13.10%
Alanine aminotransferase increased	120	12.20%
Transaminases increased	91	9.30%
Hepatic enzyme increased	77	7.80%
Platelet count decreased	49	5.00%
Troponin I increased	40	4.10%
Liver function test increased	35	3.60%
Gamma-glutamyltransferase increased	23	2.30%
Blood lactate dehydrogenase increased	22	2.20%
Body temperature increased	22	2.20%
Troponin increased	18	1.80%
Oxygen saturation decreased	15	1.50%
Troponin T increased	15	1.50%
C-reactive protein increased	14	1.40%
Heart rate increased	13	1.30%
Monocyte count increased	13	1.30%
Blood bilirubin increased	11	1.10%

**Table 5:** Adverse events reported in Zolgensma^®^-related ICSRs collected in the EudraVigilance spontaneous reporting system from 2019 to 22 September 2023, belonging to the Cardiac disorders MedDRA System Organ Class.

Adverse Events Belonging to the Cardiac Disorders MedDRA SOC (*n* = 59)	*n*	%
Tachycardia	14	23.70%
Bradycardia	11	18.60%
Cardiac arrest	9	15.30%
Cardio-respiratory arrest	4	6.80%
Tachyarrhythmia	3	5.10%
Arrhythmia	2	3.40%
Cardiac failure	2	3.40%
Pericardial effusion	2	3.40%
Pericarditis	2	3.40%
Bradyarrhythmia	1	1.70%
Cardiac disorder	1	1.70%
Cardiomegaly	1	1.70%
Myocardial hypoxia	1	1.70%
Myocardial injury	1	1.70%
Pulseless electrical activity	1	1.70%
Sinus tachycardia	1	1.70%
Toxic cardiomyopathy	1	1.70%
Ventricular extrasystoles	1	1.70%
Ventricular hypertrophy	1	1.70%

**Table 6:** Adverse events reported in Zolgensma^®^-related ICSRs collected in the EudraVigilance spontaneous reporting system from 2019 to 22 September 2023, belonging to the Hepatobiliary disorders MedDRA System Organ Class (SOC).

Adverse Events Belonging to the Hepatobiliary Disorders MedDRA SOC	*n*	%
Hypertransaminasaemia	30	25.90%
Liver disorder	10	8.60%
Acute hepatic faliure	9	7.80%
Hepatic cytolysis	9	7.80%
Abnormal hepatic function	8	6.90%
Hepatitis	8	6.90%
Hepatotoxicity	6	5.20%
Drug-induced liver injury	5	4.30%
Jaundice	4	3.40%
Cholestasis	3	2.60%
Hepatic failure	3	2.60%
Hepatomegaly	3	2.60%
Liver injury	3	2.60%
Gallbladder enlargement	2	1.70%
Hepatic fibrosis	2	1.70%
Hyperbilirubinaemia	2	1.70%
Ocular icterus	2	1.70%
Autoimmune hepatitis	1	0.90%
Cholangitis	1	0.90%
Hepatic steatosis	1	0.90%
Hepatosplenomegaly	1	0.90%
Ischaemic hepatitis	1	0.90%
Liver tenderness	1	0.90%
Subacute hepatic failure	1	0.90%

## Data Availability

EV data are publicly available at https://www.adrreports.eu/, accessed on 22 September 2023.

## Supplementary Materials

The following supporting information can be downloaded at: https://www.mdpi.com/article/10.3390/ph17030394/s1, Table S1: Other suspected drugs reported in the Individual Case Safety Reports related to Zolgensma^®^ (onasemnogene abeparvovec) collected in the EudraVigilance database from 2019 to 22 September 2023 and their distribution by therapeutic indications. Table S2: MedDRA Preferred Terms with fatal outcome reported in the Individual Case Safety Reports related to Zolgensma^®^ (onasemnogene abeparvovec) collected in the EudraVigilance database from 2019 to 22 September 2023 and their distribution by patient sex and age groups.
