# Supplementary material for: Real-World Safety Data of the Orphan Drug Onasemnogene Abeparvovec (Zolgensma®) for the SMA Rare Disease: A Pharmacovigilance Study Based on the EMA Adverse Event Reporting System
Source: Pharmaceuticals (Basel). 2024 Mar 19;17(3):394. doi: 10.3390/ph17030394 (PMC10974574; doi:10.3390/ph17030394)
Supplement: Supplementary file 1 [file pharmaceuticals-17-00394-s001.zip › pharmaceuticals-2912042-supplementary.pdf]

Supplementary Tables

Real-world safety data of the orphan drug Onasemnogene Abeparvovec (Zolgensma®) for the SMA rare disease: a pharmacovigilance study based on the EMA adverse event reporting system

Supplementary Table S1. Other suspected drugs reported in the Individual Case Safety Reports related to Zolgensma® (onasemnogene abeparvovec) collected in the EudraVigilance database from 2019 to 22 September 2023 and their distribution by therapeutic indications.

	**Overall (N=74)**
**Other Suspected Drug**	
Amoxicillin	1 (1.4%)
Ampicillin	1 (1.4%)
Calcium Gluconate	1 (1.4%)
Cefuroxime	1 (1.4%)
Cortisone	1 (1.4%)
Dexamethasone	1 (1.4%)
Famotidine	2 (2.7%)
Fentanyl	1 (1.4%)
Ibuprofen	1 (1.4%)
Lansoprazole	1 (1.4%)
Lorazepam	1 (1.4%)
Meropenem	1 (1.4%)
Methadone	1 (1.4%)
Methylprednisolone	1 (1.4%)
Midazolam	1 (1.4%)
Montelukast Sodium	1 (1.4%)
Nusinersen	23 (31.1%)
Ondansetron	1 (1.4%)
Oseltamivir	1 (1.4%)
Paracetamol	1 (1.4%)
Prednisolone	26 (35.1%)

	Overall (N=74)
Prednisone	1 (1.4%)
Ramipril	1 (1.4%)
Risdiplam	1 (1.4%)
Vitamin K	2 (2.7%)
**Therapeutic Indication**	
Gastritis Prophylaxis	1 (1.4%)
Pyrexia	1 (1.4%)
H1n1 Influenza	1 (1.4%)
Hepatic Enzyme Increased	2 (2.7%)
Hepatitis	1 (1.4%)
Hepatotoxicity	2 (2.7%)
Hypertension	1 (1.4%)
Immunomodulatory Therapy	3 (4.1%)
Immunosuppression	1 (1.4%)
In Vivo Gene Therapy	1 (1.4%)
International Normalised Ratio Increased	2 (2.7%)
Not Available	11 (14.9%)
Non Infective Gingivitis	1 (1.4%)
Product Used For Unknown Indication	23 (31.1%)
Prophylaxis	1 (1.4%)
Sinus Tachycardia	1 (1.4%)
Spinal Muscular Atrophy	20 (27.0%)
Urinary Tract Infection	1 (1.4%)

Supplementary Table S2. MedDRA Preferred Terms with fatal outcome reported in the Individual Case Safety Reports related to Zolgensma® (onasemnogene abeparvovec) collected in the EudraVigilance database from 2019 up to 22 September 2023 and their distribution by patient sex and age groups.

	**Overall (N=130)**
**MedDRA Preferred Terms**	
Abdominal sepsis	1 (0.8%)
Acute hepatic failure	4 (3.1%)
Acute kidney injury	1 (0.8%)
Acute respiratory failure	1 (0.8%)
Alanine aminotransferase increased	1 (0.8%)
Aspartate aminotransferase increased	1 (0.8%)
Aspiration	3 (2.3%)
Asthenia	3 (2.3%)
Atelectasis	1 (0.8%)
Bacterial infection	1 (0.8%)
Bilirubin conjugated increased	1 (0.8%)
Blood bilirubin increased	1 (0.8%)
Blood creatinine increased	1 (0.8%)
Blood pressure decreased	1 (0.8%)
Blood urea increased	1 (0.8%)
Bradycardia	2 (1.5%)
Brain death	1 (0.8%)
Brain injury	1 (0.8%)
Bronchial obstruction	1 (0.8%)
Cardiac arrest	8 (6.2%)
Cardiac failure	1 (0.8%)
Cardio-respiratory arrest	2 (1.5%)
Condition aggravated	1 (0.8%)

	Overall (N=130)
COVID-19	1 (0.8%)
Cyanosis	2 (1.5%)
Death	8 (6.2%)
Diarrhoea	1 (0.8%)
Disease progression	2 (1.5%)
Drug-induced liver injury	1 (0.8%)
Dyspnoea	2 (1.5%)
Emphysema	1 (0.8%)
Epistaxis	1 (0.8%)
Escherichia sepsis	1 (0.8%)
Eye colour change	1 (0.8%)
Eye movement disorder	1 (0.8%)
Gastrointestinal bacterial infection	1 (0.8%)
Gastrointestinal infection	1 (0.8%)
Gastrointestinal necrosis	1 (0.8%)
Heart rate increased	2 (1.5%)
Hepatic encephalopathy	2 (1.5%)
Hepatic enzyme increased	1 (0.8%)
Hepatic fibrosis	1 (0.8%)
Hepatic function abnormal	2 (1.5%)
Hepatitis	1 (0.8%)
Hepatotoxicity	3 (2.3%)
Hydrocephalus	1 (0.8%)
Hyperhidrosis	1 (0.8%)
Hypertension	1 (0.8%)
Hypotension	1 (0.8%)
Ill-defined disorder	1 (0.8%)
Immune system disorder	1 (0.8%)

	Overall (N=130)
Influenza	1 (0.8%)
Irregular breathing	1 (0.8%)
Jaundice	2 (1.5%)
Lethargy	1 (0.8%)
Leukocytosis	1 (0.8%)
Mass	1 (0.8%)
Metapneumovirus pneumonia	1 (0.8%)
Multiple organ dysfunction syndrome	3 (2.3%)
Ocular icterus	1 (0.8%)
Peripheral coldness	1 (0.8%)
Pneumonia	3 (2.3%)
Pneumonia aspiration	1 (0.8%)
Pyrexia	2 (1.5%)
Rebound effect	2 (1.5%)
Respiratory arrest	5 (3.8%)
Respiratory disorder	1 (0.8%)
Respiratory distress	1 (0.8%)
Respiratory failure	5 (3.8%)
Respiratory syncytial virus infection	1 (0.8%)
Secretion discharge	1 (0.8%)
Sepsis	2 (1.5%)
Soft tissue necrosis	1 (0.8%)
Spinal muscular atrophy	1 (0.8%)
Thrombocytopenia	2 (1.5%)
Toxic encephalopathy	1 (0.8%)
Transaminases increased	1 (0.8%)
Viral infection	1 (0.8%)
Vomiting	4 (3.1%)

	Overall (N=130)
**Sex**	
Female	72 (55.4%)
Male	36 (27.7%)
Not Specified	22 (16.9%)
**Age group**	
0-1 Month	1 (0.8%)
2 Months - 2 Years	123 (94.6%)
Not Specified	6 (4.6%)
